# Multidimensional relationships between sensory perception and cognitive aging

**DOI:** 10.3389/fnagi.2024.1484494

**Published:** 2024-12-20

**Authors:** Lakshmi Kannan, Esteban Sebastian Lelo de Larrea-Mancera, Marcello Maniglia, Mariya M. Vodyanyk, Frederick J. Gallun, Susanne M. Jaeggi, Aaron R. Seitz

**Affiliations:** ^1^Department of Psychology, Game Design, and Physical Therapy, Movement and Rehabilitation Services, Northeastern University, Boston, MA, United States; ^2^Department of Psychology, University of California, Riverside, Riverside, CA, United States; ^3^Department of Otolaryngology, Oregon Health & Science University, Portland, OR, United States

**Keywords:** sensory processes, cognitive aging, dementia, dual sensory loss, hearing, vision, perceptual processes

## Abstract

A growing literature suggests that declines in sensory/perceptual systems predate cognitive declines in aging, and furthermore, they are highly predictive for developing Alzheimer’s disease and Alzheimer’s related dementias (ADRD). While vision, hearing, olfaction, and vestibular function have each been shown to be related to ADRD, their causal relations to cognitive declines, how they interact with each other remains to be clarified. Currently, there is substantial debate whether sensory/perceptual systems that fail early in disease progression are causal in their contributions to cognitive load and/or social isolation or are simply coincident declines due to aging. At the same time, substantial declines in any of these senses requires compensation, can strain other neural processes and impact activities of daily living, including social engagement, quality of life, and the risk of falls. In this perspective piece, we review literature that illustrates the different relationships between sensory/perceptual systems, cognitive aging and ADRD. We suggest that broadly administered and precise assessment of sensory/perceptual functions could facilitate early detection of ADRD and pave the way for intervention strategies that could help reduce the multifaceted risk of developing ADRD and to improve everyday functioning as people age.

## 1 Introduction

The number of older adults aged 65 and above is expected to nearly double from 52 million in 2018 to 95 million by 2060 in the United States (Sleeman et al., 2019). With aging, the likelihood for developing cognitive impairments increases, with an estimated 13.9 million individuals projected to be affected by Alzheimer’s disease and related dementias (ADRD) (Tahami Monfared et al., 2022). Moreover, sensory loss, particularly hearing and vision impairment, has been linked to cognitive declines associated with aging and represent a significant issue among the aging population (Li and Lindenberger, 2002; Lindenberger and Ghisletta, 2009; Völter et al., 2021; Lee et al., 2022). It is estimated that approximately 25% of older adults will experience hearing loss (Hearing loss and hearing aid use, n.d.), 12% vision loss (American Foundation for the Blind, 2013; Prevent Blindness America, 2008), and 11% (Desai et al., 2001) both hearing and vision (dual sensory) loss. This figure rises to about 50% for those aged 75 and older. These sensory losses have significant negative impacts to quality of life; they can lead to social isolation (Mick et al., 2018; Wang et al., 2022), depression (Capella-McDonnall, 2005; Capella, 2009), and a higher risk of falls (Völter et al., 2021; Lindenberger and Baltes, 1994; Wilson et al., 2007). Critically, declines in sensory/perceptual systems have also been shown to be predictive for developing ADRD (Livingston et al., 2024), although the underlying mechanisms and causal relationships are still poorly understood. Thus, there is a great need for research that better characterizes relationships between sensory loss, age-related cognitive declines, and ADRD, as well as for clinical tools that can help with early detection, prevention, and appropriate management strategies to reduce the burden on the healthcare system and society at large.

Currently, the relationship between sensory loss and cognitive function is supported by two predominant theories: cascade theory and common cause theory (Masten and Cicchetti, 2010). The cascade theory postulates that increased cognitive load due to impaired perceptual processes (Anstey et al., 2001; Lin et al., 2011; Livingston et al., 2017; Wayne and Johnsrude, 2015), adds strain to cognitive systems (Zekveld et al., 2011; Martini et al., 2015; Humes and Young, 2016; Monge and Madden, 2016; Roberts and Allen, 2016; Schieber, 2003; Martini et al., 2015), leading to impaired performance. Further, there is a significant association of sensory loss with difficulty conducting complex tasks (Veldkamp et al., 2021; McIsaac et al., 2018; Danneels et al., 2023; Wunderlich et al., 2024), increased social isolation (Mick et al., 2018; Wang et al., 2022), and reduced independence (Raina et al., 2004), which might further contribute to the development of ADRD (Livingston et al., 2024). While the brain is capable of compensating for sensory losses to a certain degree, the associated neuroplasticity could become maladaptive resulting in structural and functional impairments, contributing to cognitive decline (Seidler et al., 2010; Slade et al., 2020; Cardin, 2016). Alternatively, the common cause theory assumes that both sensory and cognitive impairments are interconnected with atrophy and pathophysiological changes of the brain as a result of aging. Both theories explain the link between sensory deprivation and dementia risk, and they are often considered as overlapping concepts rather than mutually exclusive (Mahmoudi, 2021).

**Figure 1:**
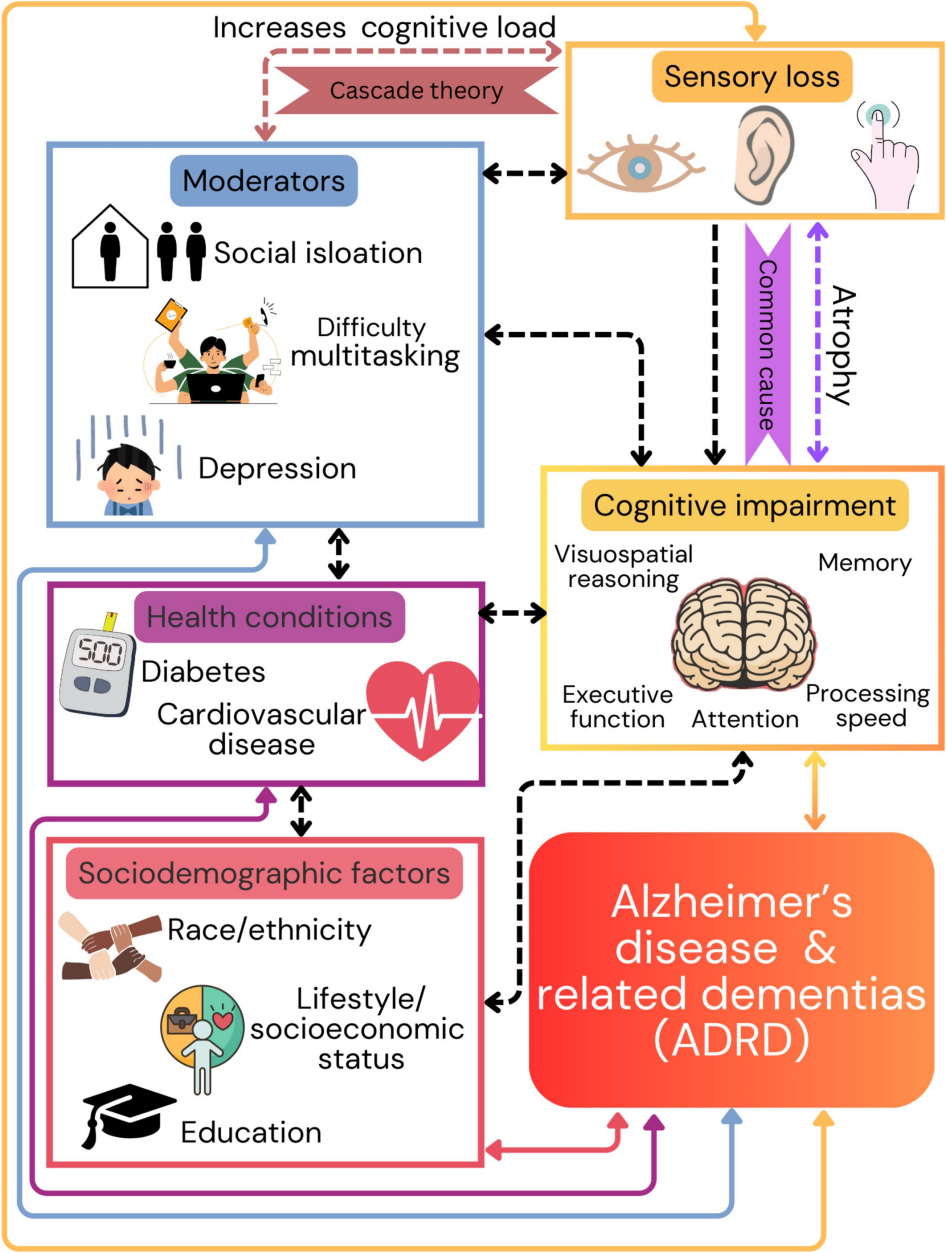
This figure illustrates the multifactorial nature of dementia risk, emphasizing the importance of addressing sensory health and cognitive function, along with social and health-related factors, to mitigate the onset and progression of dementia and Alzheimer’s disease related dementias (ADRD). Sensory loss within visual, auditory, and tactile processes is associated with the negative impact on higher cognitive functions based on two theories (cascade and common cause). Additionally, sensory loss contributes to social isolation, reduced independence, and other health conditions including high blood pressure, cardiovascular diseases, and diabetes. Arrows from these factors point towards the bottom of the figure, indicating their cumulative contribution to the risk of developing ADRD.

The interrelationship between sensory loss and cognitive decline in aging involves complex mechanisms that span biological, psychological, and social domains (Wayne and Johnsrude, 2015; Pichora-Fuller et al., 2015; Baltes and Lindenberger, 1997; Li and Lindenberger, 2002; Baltes and Lindenberger, 1997). Further, research suggests that several modifiable risk factors interact and impact cognitive aging which include high blood pressure, cardiovascular diseases, and diabetes. These individualized risk factors often occur already in middle age (Mahmoudi, 2021; Kivimäki et al., 2019; Fayosse et al., 2020), suggesting that early prevention and intervention could have long-lasting impacts. Further, sociodemographic factors such as race/ethnicity, lifestyle, socioeconomic status, and educational attainment also impact ADRD risk (Mahmoudi, 2021; Kivimäki et al., 2019; Fayosse et al., 2020). However, although numerous associations between sensory loss and ADRD have been demonstrated, causal relationships have yet to be unambiguously demonstrated (Dawes et al., 2015), and the understanding of whether and how they interact with modifiable risk and sociodemographic factors is still limited (Figure 1).

To better understand the relationships between sensory loss and cognitive function, there is a need for valid, reliable, and cost-effective measures that are sensitive enough to capture early sensory and cognitive declines. Furthermore, establishing causal relationships requires interventional and/or longitudinal data, which can be difficult to acquire due to extended timeframes. Yet, current research practices often fail to address central perceptual processes despite their clinical relevance. For example, assessments of low-level (e.g., basic sensory acuity tests) and mid-level (e.g., perceptual organization tasks) perceptual processing can provide fundamental information regarding people’s perceptual functions. While there are numerous tests of central perceptual processes, many of these are restricted to use in basic research and are rarely adopted for large-scale clinical use. There are multiple limitations of existing assessments of sensory and cognitive function that restrict translation to clinical practice and their implementation at scale. These include (1) requirement for specialized equipment and associated costs, (2) substantial time required for measurement, (3) lack of standardization, (4) lack of normative data on sensitivity and specificity of the different measures to predict cognitive decline and ADRD, and (5) lack of accessible assessment tools that include the latest advances in both theory and technology. In the following sections, we will review approaches to measure sensory/perceptual processes (with an emphasis on hearing and vision), and discuss opportunities to improve practice and implementation.

### 1.1 Hearing and cognition

Pure tone audiometry, used to establish the quietest sounds that a person can hear at different frequencies, constitutes the gold-standard clinical assessment of hearing (Musiek et al., 2017; Eberhard et al., 2023). Elevated hearing thresholds captured by these clinical audiograms are the traditional definition of hearing loss, affecting 20% of people worldwide (WHO, 2021). They are thought to represent declines in the peripheral auditory system. The risk for this peripheral hearing loss increases with age (Huang and Tang, 2010), with over 60% of all individuals with hearing impairment being older than 50, and over 50% of people experiencing at least a moderate level of peripheral hearing loss by the age of 90 (Haile et al., 2021). Meta-analytic work using large-scale longitudinal studies investigating the relationship between peripheral hearing loss and cognitive decline (Lin et al., 2011; Zheng et al., 2017; Idrizbegovic et al., 2013; Häggström et al., 2019) has identified peripheral hearing loss in mid-life as a major modifiable risk factor, predicting the development of dementia later in life (Livingston et al., 2017; Livingston et al., 2020; Nianogo et al., 2022; Liang et al., 2021; Litke et al., 2021; Omura, 2022). Importantly, peripheral hearing loss can be detected years before the clinical manifestation of dementia (Gates et al., 2002; Ralli et al., 2019; Albers et al., 2015), suggesting that peripheral hearing loss could serve both, as a critical early marker and a target for intervention (Nagaraj, 2024; Powell et al., 2022; Thomson et al., 2017; Powell et al., 2021).

Although pure tone audiometry is standardized and has clear clinical relevance, there is a high prevalence of individuals with relatively normal audiograms, but who experience and self-report great hearing difficulties, in particular, understanding speech in noisy conditions, such as following a conversation in a restaurant (Shub et al., 2020; Stiepan et al., 2020; von Gablenz and Holube, 2017; Smoorenburg, 1992; Lee et al., 2015; Koerner et al., 2020). Furthermore, clinical categories such as hidden hearing loss (Liberman et al., 2016) or central auditory processing disorder (Sardone et al., 2020; Sanchez et al., 2008) are well-established and illustrate that hearing difficulty can be only partially assessed with pure tone detection measures. Contributions to hidden hearing loss are manifold where a deficit or disconnection in any part of the ascending auditory pathways, from the cochlea to the brainstem and midbrain to the auditory cortices and onward to frontal and parietal regions, can impair auditory processing (Felix et al., 2018). Central auditory processing functional deficits associated with age [e.g., central presbycusis (Mazelová et al., 2003; Humes et al., 2012)] can include difficulties in processing rapidly changing sounds (Bernstein et al., 2013; Grose and Mamo, 2010; Grose and Mamo, 2012; Mehraei et al., 2014; Ozmeral et al., 2016), locating sounds in space (Bremen and Middlebrooks, 2013; Eddins et al., 2018; Hoppe et al., 2022; Stecker and Gallun, 2012), attending to sounds of interest (Gallun and Best, 2020), processing of contextual cues that guide meaning (Bremen and Middlebrooks, 2013; Hoppe et al., 2022; Gallese and Lakoff, 2005; Kuhl and Damasio, 2012), and understanding speech in noisy conditions (Danhauer et al., 2018). To successfully understand speech, we need to detect and discriminate rapid changes in the temporal and spectral structures of broad-band sounds in complex auditory scenes (Oxenham, 2018; Parise et al., 2014; Bregnian, 1993; Snyder et al., 2012), where speech signal needs to be segregated from the competition to be successfully perceived (Gallun and Jakien, 2019; Ding and Simon, 2012). In addition, cognitive processes including attention and working memory also contribute to speech-in-noise comprehension (Gallun and Best, 2020; Bronkhorst, 2015; Miyake and Shah, 1999).

While complaints of not being able to hear conversational partners are ubiquitous among older adults (Humes, 2021), assessments of central auditory processes are not broadly administered, despite the fact that self-reported hearing difficulties may be better explained by speech-in-competition measures than the audiogram (Lelo de Larrea-Mancera et al., 2024). Further, there is growing evidence that central auditory processes may be predictors of ADRD, but that relationship remains largely understudied. For example, Gates et al. (2011) and Mohammed et al. (2022) found that the Dichotic Sentence Identification test, a standard measure of speech-in-competition, was associated with elevated risk for dementia (up to 4 times), even after controlling for pure tone audiogram thresholds. However, there are no gold-standard tests to measure self-reported hearing ability, speech-in-competition, or other central auditory processes. Thus, there is a substantial need to establish and standardize measures of hearing to accurately measure possible dysfunction at different stages of auditory processing and establish their relationships to age-related cognitive decline and ADRD.

### 1.2 Vision and cognition

Measures of visual acuity, used to determine the smallest characters a person can read, currently serve as the gold standard of assessing visual abilities. The Snellen chart, introduced in 1862, is the earliest and best-known standardized visual acuity measurement tool, It consists of letters arranged in rows of decreasing size (Azzam and Ronquillo, 2020; Rozhkova et al., 2021).

Visual impairment, as captured with low-level measures such as visual acuity, can be caused by several factors, including altered visual inputs of optic or retinal nature, or impairment of cortical perceptual processing. For example, in macular degeneration - the most common cause of visual impairment in the western world - degeneration of foveal photoreceptors leads to central vision loss. This conditions greatly compromise everyday tasks such as reading, navigating, and recognizing faces. However, losing central vision deprives patients not only of their retinal location with the highest resolution, but also it impacts their oculomotor attentional processes (Sabbah et al., 2017; Zhuang et al., 2021). The relationship of the visual system with higher level processing regions is reflected in the tight connection between visual and cognitive impairment, with multiple studies showing that macular degeneration and Alzheimer’s disease share common risk factors and histopathological changes. Furthermore, there is epidemiological evidence linking macular degeneration to cognitive impairment (Livingston et al., 2020; Johnson et al., 2002; Kaarniranta et al., 2011) including impaired performance in tasks of memory, executive functions, and global cognition (Rozzini et al., 2014).

Studies that provide causal evidence for the relationship between vision loss and ADRD are at its infancy. In regard to the common cause model, tests using degraded visual letters show promise to detect visual Alzheimer’s disease (Yong et al., 2024). In regard to the cascade theory, recent investigations have demonstrated that cataract surgery, an extremely common visual restoration procedure, is associated with amelioration of age-related cognitive declines (Fukuoka et al., 2016; Jefferis et al., 2015; Jefferis et al., 2013). Similarly, in glaucoma, a neurodegenerative condition affecting both visual and cognitive functions, strategies such as vision rehabilitation (i.e., visual and cognitive training) (Li et al., 2020; Patodia et al., 2017; Livengood and Baker, 2015; Sabel and Gudlin, 2014; Deemer et al., 2023), assistive technologies (screen readers or text-to-speech software) (Vice, 2024; Whittaker et al., 2024; Sarma et al., 2018; Trpcheski and Chorbev, 2022), and pharmacological drugs (e.g., eye drops) (Shalaby et al., 2020) have shown potential benefits on improving cognitive performance, suggesting that visual impairment could be a modifiable risk factor for pathological cognitive decline. Although visual acuity is an important measure of vision function, it is only one of many different visual processes that can be relevant to understand visual impairments. As with hearing, there are numerous stages of visual processing that are all important to functional vision. For example, our ability to read words or recognize faces, depends on our ability to process small changes in luminance contrast, relative depths, and motion, all of which help us detect important visual features that we integrate into objects and then process these using higher cognitive functions including attention and memory. Visual impairments related to higher-level visual functions are linked to reduced quality of life and negative psychological well-being.

### 1.3 Dual sensory loss

While it is common for research on sensory impairments to focus on single conditions, dual sensory loss refers to cases where impairments impact multiple senses. For example, Desai and colleagues conducted an epidemiological study that suggested that at least 11% of the population experiences dual hearing and vision loss, and most people express concerns of both hearing and vision as they age (Desai et al., 2001). Furthermore, there is evidence that the impacts of dual sensory loss accelerates the risk of developing ADRD, compared to those without sensory impairments (Ge et al., 2021). In line with this, other work has shown that people with dual sensory loss are twice as likely to develop ADRD than those without any sensory impairments (Hwang et al., 2020).

However, dual sensory loss often remains undetected due to fragmented healthcare systems (i.e., the division of audiology and optometry) and the prioritization of more acute medical conditions. Thus, in addition to the need to better characterize our individual senses, it is also important to measure how they work together. For example, tests of audiovisual integration (de Dieuleveult et al., 2017; Freiherr et al., 2013), can help us understand how sensory information from multiple modalities interact. In addition, tests like the audio-visual divided attention task, where attention must select between, or be divided among sensory streams can help us understand better how sensory systems can also compete for attentional and memory resources (Koerner et al., 2024). Further, it is important to understand relationships with sense beyond just hearing and vision. For example, vestibular functions that have strong impacts on mobility processes such as balance control impairments and vestibular dysfunction leading to falls, also interact with both, hearing and vision, as well as higher cognitive functions, and ultimately, predict ADRD (Agrawal et al., 2020; Wei et al., 2019).

## 2 Recommendations

As highlighted in the sections above, sensory/perceptual loss is a multidimensional set of conditions that has complex, and likely insufficiently understood relationships with cognition and ADRD. Our current medical system has traditionally separated clinical approaches to treat hearing loss, vision loss, as well as evaluations and treatments for motor and cognitive function, which has systematically limited the research required to understand how co-occurring declines across all these systems interact with aging (another separate medical discipline), and ADRD. While it is obvious to all of us that with age, our vision, hearing, mobility, as well as cognition decline, however, a better understanding of how these systems interact to impact our long-term health and wellbeing requires a paradigm shift and creation of new standards and practices.

For example, while existing standards such as pure tone audiometry for hearing and acuity tests for vision do provide useful functional evaluations that lead to easily deployable interventions such hearing aids and vision aids (e.g., glasses, contacts), these assessments fall short in capturing the complex, real-world sensory impairments and cognitive interactions that affect everyday life. A tangible example would involve engaging in a simple conversation with friends at a restaurant that involves complex interactions between central auditory processing to select and processing the voices of our conversational partners over the many distracting noise sources. These processes are facilitated by our visual system that can view our friends’ lips, and multisensory processing to integrate this information to understand their speech. Lastly, encouraging simultaneous performance of more than one task (cognitive-motor, motor-motor, cognitive-cognitive) as we process other relevant visual information related to our food and drinks, and coordinate with our motor system to bring food to our mouth. All these examples further tap into cognitive resources involving attention, memory and cognitive control processes related to speech comprehension.

To address the complex, multiple domain activities that are ubiquitous of daily functions, we need new paradigms that better address the dimensionality and complexity of sensory/perceptual and cognitive processes that people rely upon, and to understand the individual trajectories of these, and their interactions with aging processes. These can be facilitated by advancements in consumer technology, peoples’ phones and computers, and even more impressively, new extended reality (XR) systems that have more advanced audio and visual processing systems that are found in many high-end clinical evaluation systems. This gives rise to transformative potential to advance both in-patient and out-patient assessments of sensory/perceptual and cognitive processes. Further, with gyroscopes and accelerometers becoming increasingly standard in smart-phone, smart watches, or XR headsets, it could be convenient to evaluate postural control (King et al., 2024; King et al., 2017; Martini et al., 2024; Campbell et al., 2023; Mancini et al., 2012; Fino et al., 2017; Kelly et al., 2022; Murray et al., 2023) in simple single as well as dual tasking conditions that can help understand dual models of cognitive decline (Tian et al., 2023) among others.

Building on these technological advancements highlights the transformative potential of low-cost consumer technologies for home-based clinical screening (Coppola et al., 2024). For example, it is easy for someone to conduct a large battery of tests of both peripheral and central processing with a high degree of fidelity. In the case of hearing, tests of spatial release from masking (Pastore and Yost, 2017), where one must listen to the instructions of one speaker while ignoring other people speaking similar but misleading instructions, can provide a measure of hearing in complex, real-world-like environments. Digital technology also allows for convenient testing of hearing handicap, pure-tone sensitivity, spectral, temporal, and spectral-temporal processing, and other speech in competition tests in at-home settings (Lelo de Larrea-Mancera et al., 2022), and thus, improving the tests’ real-world relevance. Similarly, in the case of vision, complex tasks such as trail making or visual search can be digitally administered and followed-up by tests of more basic visual functions such acuity, contrast and color sensitivity, stereo vision, and motion processing (Jayakumar et al., 2024). These measures can be complemented with standard neuropsychological tasks capturing memory, executive functioning, language, and visuospatial reasoning, and at the same time, their digital administration can reveal data that go beyond traditional paper-pencil tests that have dominated standard cognitive evaluation. Furthermore, the integration of motion sensors in these devices facilitates the estimation of postural control, which is crucial for understanding cognitive decline through dual-tasking conditions and dual sensory loss (Kannan et al., 2024; Pitts et al., 2023; Mancini et al., 2012; Fino et al., 2017). To fully leverage these advancements however, the development of normative data and standardized protocols is essential, ensuring accuracy and reliability across various settings (Campbell et al., 2023). Utilizing widely available and low-cost consumer devices also improves accessibility and efficiency, allowing for quicker and more frequent evaluations, which is particularly beneficial for early detection, monitoring progressive conditions, and adjusting treatments promptly (Murray et al., 2023). As such, digital assessments can provide a unique opportunity to advance our understanding of the multidimensional relationships between sensory/perceptual functions and cognition in ADRD, however, it will be important to address key challenges such as standardization, time efficiency, and accessibility.

## Data Availability

The original contributions presented in the study are included in the article/supplementary material, further inquiries can be directed to the corresponding authors.
